# Neuromechanical control of impact absorption during induced lower limb loading in individuals post-stroke

**DOI:** 10.1038/s41598-022-21271-8

**Published:** 2022-11-09

**Authors:** Keng-Hung Shen, Sunil K. Prajapati, James Borrelli, Vicki L. Gray, Kelly P. Westlake, Mark W. Rogers, Hao-Yuan Hsiao

**Affiliations:** 1 Department of Kinesiology and Health Education, University of Texas at Austin, Austin, TX USA; 2 Department of Applied Physiology and Wellness, Southern Methodist University, Dallas, TX USA; 3 Department of Physical Therapy and Rehabilitation Science, University of Maryland, Baltimore, MD USA; 4 Department of Biomedical Engineering, Stevenson University, Baltimore, MD USA

**Keywords:** Health care, Medical research, Neurology

## Abstract

Decreased loading of the paretic lower limb and impaired weight transfer between limbs negatively impact balance control and forward progression during gait in individuals post-stroke. However, the biomechanical and neuromuscular control mechanisms underlying such impaired limb loading remain unclear, partly due to their tendency of avoiding bearing weight on the paretic limb during voluntary movement. Thus, an approach that forces individuals to more fully and rapidly load the paretic limb has been developed. The primary purpose of this study was to compare the neuromechanical responses at the ankle and knee during externally induced limb loading in people with chronic stroke versus able-bodied controls, and determine whether energy absorption capacity, measured during induced limb loading of the paretic limb, was associated with walking characteristics in individuals post-stroke. Results revealed reduced rate of energy absorption and dorsiflexion velocity at the ankle joint during induced limb loading in both the paretic and non-paretic side in individuals post-stroke compared to healthy controls. The co-contraction index was higher in the paretic ankle and knee joints compared to the non-paretic side. In addition, the rate of energy absorption at the paretic ankle joint during the induced limb loading was positively correlated with maximum walking speed and negatively correlated with double limb support duration. These findings demonstrated that deficits in ankle dorsiflexion velocity may limit the mechanical energy absorption capacity of the joint and thereby affect the lower limb loading process during gait following stroke.

## Introduction

Over 795,000 people are affected by stroke every year in the United States, which resulted in nearly $53 billion in overall costs between 2017 and 2018^1^. Approximately one in three stroke survivors experience long-term disability, which makes stroke one of the leading causes of disability in the United States. Among people with hemiparesis due to stroke, reduction in magnitude, duration, and rate of loading of the paretic lower limb during functional activities are commonly observed and persists after the injury. For example, individuals with chronic stroke commonly bear less weight on the paretic leg than the non-paretic leg during quiet standing, sit-to-stand, and walking^2–4^, have shorter paretic single stance time than non-paretic during walking^5^, and take longer duration to transfer body weight from the non-paretic limb to the paretic limb during walking^4^. Deficits in paretic lower limb loading are associated with higher postural sway, lower overall motor function, and an increased incidence of falling^6–10^. Improvements in loading capacity of the paretic lower limb are also associated with increased balance control, walking speed, and reduced risk of falls^11–13^. In addition, the ability to transfer body weight between limbs is an important biomechanical factor that relates to limb loading^14^. Longer duration and decreased amplitude of weight transfer from the non-paretic leg to the paretic leg have been reported in individuals post-stroke that negatively impacts forward progression^6,15^. Thus, improving weight transfer and paretic lower limb loading ability are major focuses in post-stroke rehabilitation.

Different approaches have been attempted to restore paretic limb loading and weight transfer ability post-stroke. For example, de Haart and colleagues reported that during standing, loading asymmetry between the lower limbs was reduced after 12 weeks of conventional physical therapy^16,17^. Another study used a shoe insole installed beneath the non-paretic leg to shift the body center of mass toward the paretic side during daily activity^18^. After 6 weeks of interventions focused on weight shifting, significant improvements in lower limb loading symmetry along with increased walking speed were found^13,18,19^. Overall, these findings suggest that individuals post-stroke have the capacity to improve paretic lower limb loading function. However, despite the moderate improvement achieved with these interventions, previous results generally failed to show long-term success of the recovery^16,20^. This is partly due to the limited understanding of biomechanical and neuromuscular control mechanisms underlying impaired paretic limb loading ability.

During movements such as walking, landing, and protective stepping, functional limb loading requires the lower extremity to regulate the impact force and absorb or dissipate the kinetic energy^21–23^. In particular, the ankle and knee joints are the primary impact energy absorbers during rapid limb loading^24,25^ and therefore contribute to a smooth weight transfer process^22^. During the double support phase of gait, body weight is normally transferred from the trailing limb to the leading limb, requiring a coordinated push-off work from the trailing limb and corresponding energy absorption at the leading limb to ensure an efficient step-to-step transition^26,27^. Thus, deficits in energy absorption ability may disrupt the weight transfer process and ultimately impact walking speed.

Using a novel perturbation to impose rapid loading towards the paretic lower extremity, we have previously identified stroke-associated abnormalities in ankle and knee joint kinematics^28^. Other studies have reported increased joint stiffness and co-contraction of the agonist and antagonist muscles at the ankle and knee joints during voluntary movements in individuals post-stroke compared to neurologically intact adults^29–33^. These observations suggested potential neuromechanical factors underlying impaired limb loading and weight transfer following stroke. However, whether and how these neuromechanical factors affect energy absorption during rapid limb loading following stroke remains unclear. Furthermore, the relationships between energy absorption ability during perturbation-induced limb loading and functional gait characteristics remain to be determined.

Accordingly, the primary purpose of this study was to compare the rate of energy absorption and agonist–antagonist muscle co-contraction at the ankle and knee joints during a perturbation-induced limb loading in individuals post-stroke versus age-matched controls. In addition, we aimed to determine whether the rate of energy absorption measured during induced limb loading assessment was associated with walking characteristics in individuals post-stroke. We hypothesized that: (1) Compared to healthy age-matched controls, reduced rate of energy absorption and increased co-contraction of the agonist and antagonist muscles at ankle and knee joints would be observed in people with chronic stroke; and that (2) the rate of energy absorption (i.e. power) at ankle and knee joints during induced limb loading would negatively correlate with double support duration during walking and positively correlate with gait speed in people with chronic stroke.

## Methods

### Participants

Fifteen individuals post-stroke (62.8 ± 8.0 years; six females; time post-stroke 13.5 ± 12.7 years; three right paretic) along with fifteen age-matched healthy controls (64.2 ± 8.2 years; six females; 14 right dominant) participated in the study. The inclusion criteria of individuals post-stroke were (1) more than 6 months since last stroke, (2) ability to walk 10 m with or without a walking aid, and (3) ability to stand without any support for 5 min. Able-bodied controls were included if they had no self-reported history of any neurological injuries or any current musculoskeletal conditions. Exclusion criteria for both groups were: (1) underlying medical history precluding participation in regular exercises, (2) unable to follow instructions, and (3) self-reported pregnancy. The study was approved by the University of Maryland Baltimore Institutional Review Board and all participants provided written informed consent to participate. All experiments were performed in accordance with relevant guidelines and regulations.

In the final analysis, data for one participant with stroke were excluded due to technical issues in EMG recording. In addition, because we were primarily focusing on the biological factors that affect limb loading, data for two additional participants with chronic stroke who wore an ankle–foot orthosis (AFO) that may affect ankle joint movement were excluded (remaining N = 12 for stroke, N = 15 for control; Table 1).

**Table 1:** Subject characteristics in both groups.

Stroke						
ID	Sex	Age	Years post-stroke	Paretic side	Self-selected walking speed (m/s)	Maximum walking speed (m/s)
ULD01	M	58	15	L	1.06	1.43
ULD02	F	61	11	L	0.86	1.12
ULD03	M	70	10	L	0.57	0.75
ULD04	M	67	9	L	1.05	1.27
ULD05	F	76	54	L	0.80	1.03
ULD06	M	56	15	L	0.89	1.30
ULD07	M	64	1	L	0.54	0.80
ULD09	F	53	3	R	1.28	1.58
ULD11	M	53	8	L	0.88	1.02
ULD12	M	78	20	R	1.43	1.65
ULD13	F	64	22	L	0.71	1.07
ULD16	M	51	2	R	0.72	1.50
Mean ± std		62.6 ± 8.9	14.2 ± 14.2		0.90 ± 0.27	1.21 ± 0.29

Control						
ID	Sex	Age	Dominant side			
ULD51	M	56	R			
ULD52	F	63	R			
ULD53	M	78	R			
ULD54	F	56	R			
ULD55	F	73	L			
ULD56	F	65	R			
ULD57	M	67	R			
ULD58	M	62	R			
ULD59	M	65	R			
ULD60	F	67	R			
ULD61	F	53	R			
ULD62	M	77	R			
ULD63	M	62	R			
ULD64	M	50	R			
ULD65	M	69	R			
Mean ± std		64.2 ± 8.2				

### Testing procedure

Participants with chronic stroke were instructed to walk at their self-selected and then maximal walking speeds along an instrumented 7-m unobstructed GAITRite walkway (CIR Systems Inc., NJ, US).

Next, an imposed limb loading assessment was performed by using two adjacent perturbation platforms (height ~ 37 cm) each placed above a force platform (Advanced Mechanical Technology Inc., Watertown, MA). Each of the perturbation platforms included a detachable standing support surface held securely on top of a support frame using ten electromagnets (12 V DC, Magnetech Corp.). Disengagement of the magnets via a computer-controlled external circuit released the detachable surface causing it to drop 4.3 cm vertically. A layer of noise-reduction material (thickness ~ 5 mm) was glued at the bottom of the detachable surface to minimize a sound induced startle response, which is commonly triggered by a sudden acoustic stimulation over 70 dB^34^. From our preliminary result, the drop distance of 4.3 cm was capable of inducing a peak loading of approximately 90% body weight on the dropped lower limb without causing safety concerns.

**Figure 1:**
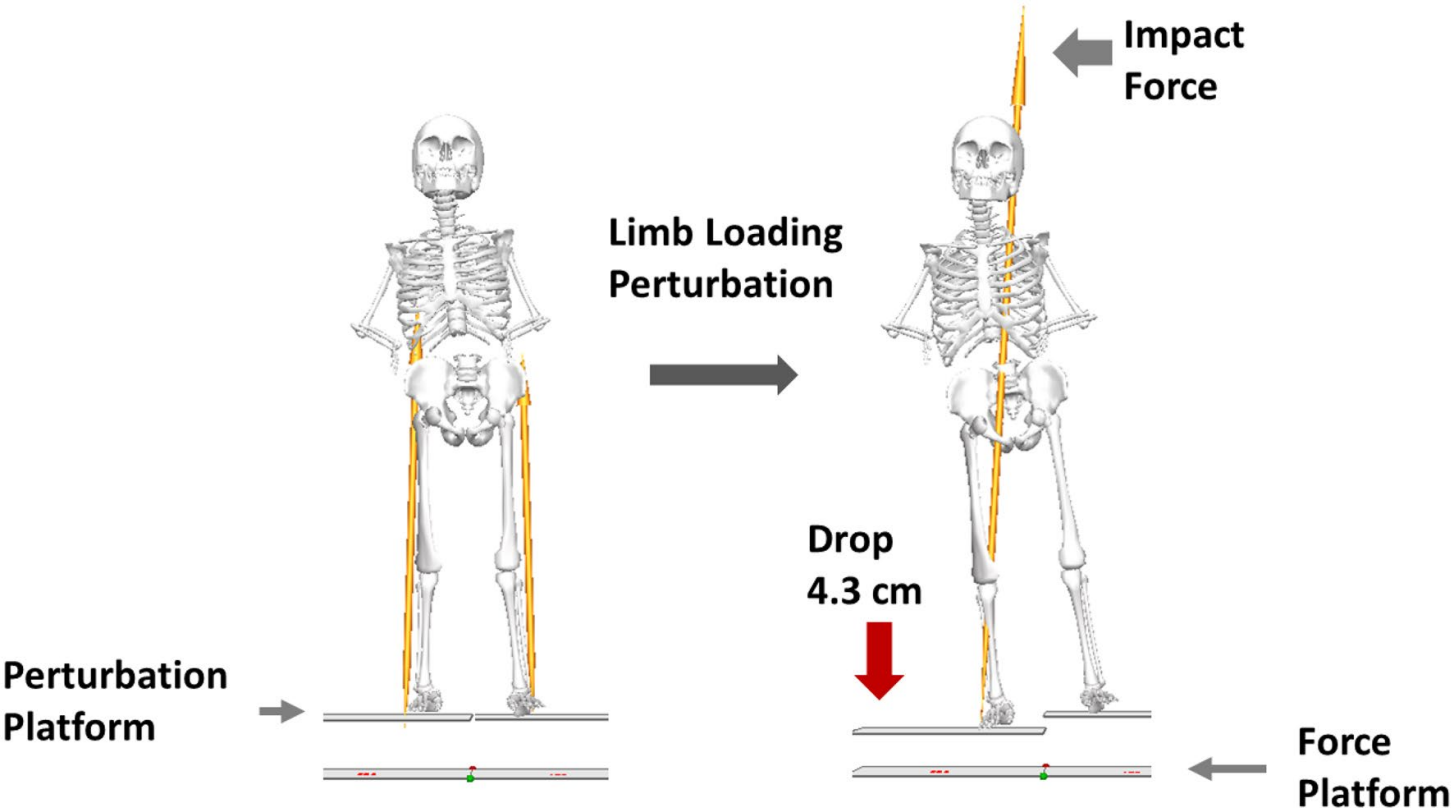
Schematic diagram of the imposed limb loading perturbation. The figure is generated using Visual3D (C-motion, inc., MD, US) and Microsoft PowerPoint (Microsoft, WA, US).

Participants stood with one foot on each perturbation platform and were instructed to stand naturally with their weight evenly distributed on each leg. The vertical ground reaction forces were monitored to ensure symmetrical limb loading prior to perturbation onset. During each trial, one of the detachable surfaces was released at an unpredictable time, causing a sudden body weight transfer towards the dropped side and induced loading of the perturbed lower limb (Fig. 1). Participants were not aware of the side of perturbation and were instructed to react to the perturbation naturally and to maintain an upright posture. Participants were not allowed to hold onto anything during the imposed limb loading assessment. Two familiarization trials (one for each leg) were performed. Next, participants completed eight trials consisting of four perturbations delivered to each leg in a randomized order. The position of the feet on the platform was marked to ensure a consistent foot positioning throughout all trials.

### Data recording

Kinematic, kinetic, and electromyographic signals were only recorded during the imposed limb loading assessment and not during gait assessment. A 10-camera Vicon motion capture system (Oxford Metrics, Oxford, UK) was used to capture the 3-dimensional body segment position. Plug-in Gait marker set^35^ were applied with additional markers attached bilaterally on the 5th metatarsal, medial elbow, greater trochanter, medial knee, medial malleoli, and the detachable surfaces of the perturbation platform. Marker data were sampled at 150 Hz for 7 s. Two force platforms (Advanced Mechanical Technology Inc., MA, US) each located beneath a perturbation platform were used to record ground reaction forces (GRF) and center-of-pressure (COP) data. Force data were sampled at 1500 Hz for 7 s. Muscle activation patterns were recorded bilaterally with surface electrodes (Noraxon, Inc., Scottsdale, AZ) placed over the gluteus medius (GM), vastus lateralis (VL), biceps femoris (BF), medial gastrocnemius (MG), and tibialis anterior (TA) muscles. EMG signals were sampled at 1500 Hz for 7 s.

### Data analyses

Overground walking speed, double support duration (% gait cycle) when the paretic limb was the leading limb, and double support duration (% gait cycle) when the non-paretic limb was the leading limb were calculated by the GAITRite software during both self-selected and maximum speed conditions.

Kinematic and kinetic data were low pass filtered at a cutoff frequency of 6 and 30 Hz, respectively. EMG signals were bandpass-filtered at 16–500 Hz, de-meaned, full-wave rectified, and lowpass-filtered at 20 Hz. All the data were processed offline with a fourth-order Butterworth filter.

**Figure 2:**
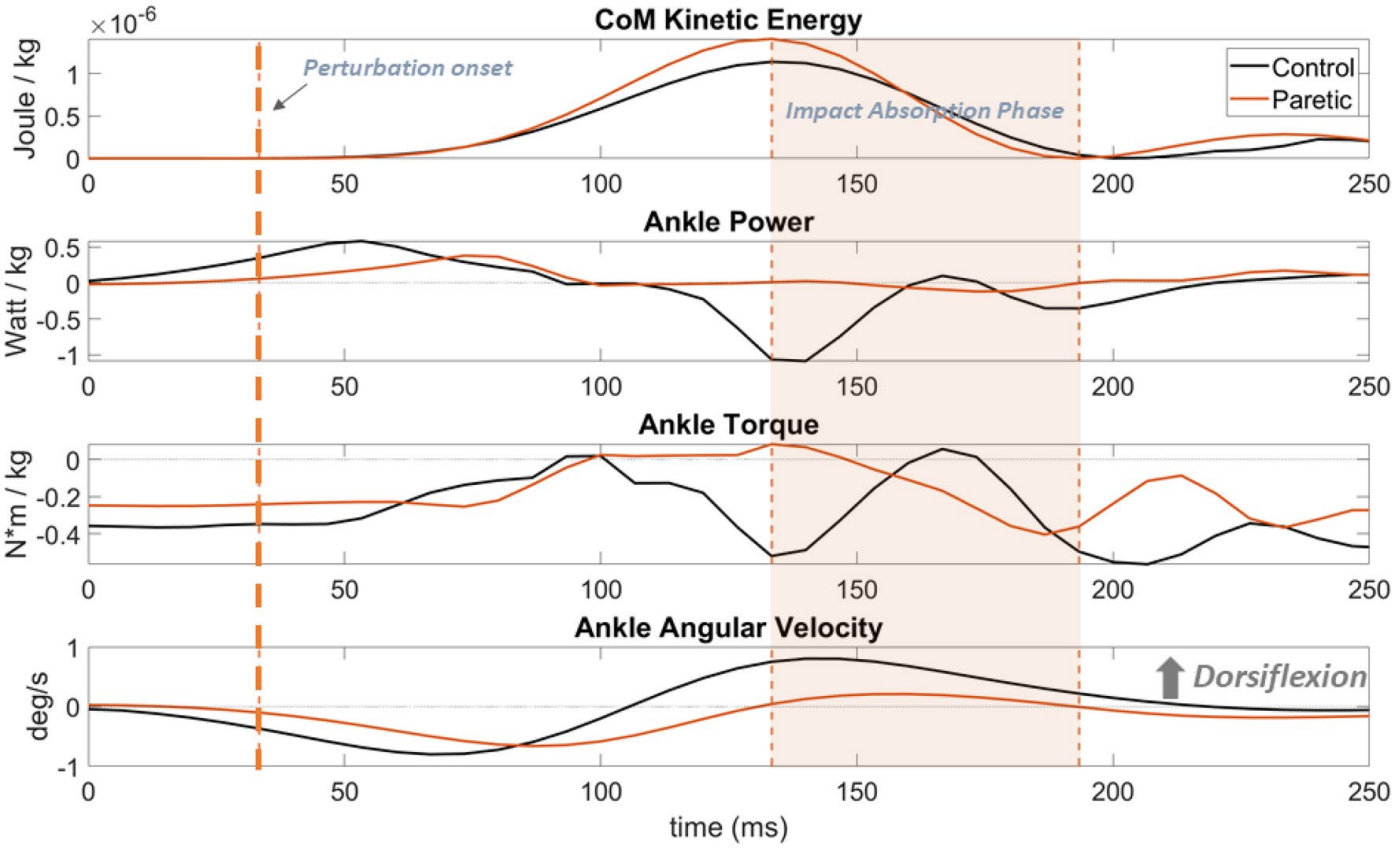
A representative trial showing CoM kinetic energy and ankle joint power, torque and angular velocity from a control participant and the paretic side of a participant post-stroke. Highlighted area represents the impact absorption phase for the paretic limb.

A custom 15 segment model (head, trunk, pelvis, and bilateral foot, shank, thigh, upper arm, forearm and hand) was used to calculate body center of mass (CoM) position, sagittal plane ankle and knee joint angle, torque and power using Visual3D software (C-motion, inc., MD, US). Downward kinetic energy of the CoM and ankle and knee joint angular velocity were then calculated. The ground reaction forces, joint torque and power, and kinetic energy were all normalized to body mass. Because we were interested in the interval when the downward kinetic energy was absorbed, the impact absorption phase was defined as the time period from the first maximum downward kinetic energy of CoM to the first minimum downward kinetic energy of CoM following a perturbation^36^ (Fig. 2). Peak values of the joint angular velocity and torque, and the minimum values of the joint power (i.e. maximum negative power) were extracted from the impact absorption phase. Since the negative value of joint power represents absorption of the energy, absolute values of minimum joint power were defined as peak rate of energy absorption. All calculations were performed with a customized program in MATLAB (The MathWorks, Inc., MA, US).

Maximum EMG amplitude within 1 s after perturbation onset was identified for each muscle. The maximum value observed across all four trials was used for normalization^37^. Muscle co-contraction was calculated using Eq. (1)^38^. This method has been shown to better reflect joint stiffness among other commonly used measurements of muscle co-contraction^39^.

1 $${\text{Co}} - {\text{contraction}} = { }\frac{{{\text{lower EMG}}_{{\text{i}}} }}{{{\text{higher EMG}}_{{\text{i}}} }}{ } \times { }({\text{lower EMG}}_{{\text{i}}} + {\text{higher EMG}}_{{\text{i}}} ).$$

**Figure 3:**
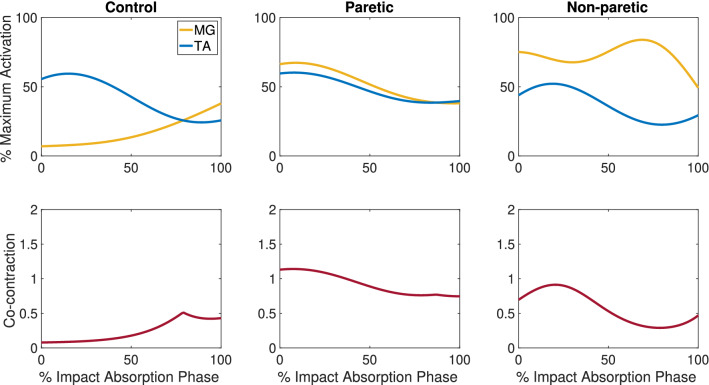
Representative trial of ankle muscle activation in the paretic and non-paretic side post-stroke and control during the impact absorption phase. The normalized EMG signals are shown in the upper panels, and the lower panels represent co-contraction duration during the impact absorption phase. *MG* medial gastrocnemius, *TA* tibialis anterior. Co-contraction was calculated with Eq. (1) between MG and TA.

Co-contraction was calculated between an agonist–antagonist pair, and “lower EMG_i_” represents the muscle that had lower EMG amplitude at time point, i, while “higher EMG_i_” represents the muscle that had higher EMG amplitude at time point, i. Co-contraction was calculated at each time point during the baseline (100 ms to 0 ms prior to perturbation onset) and the impact absorption phase (Fig. 3). Next, the time series value was integrated throughout the entire phase to obtain the co-contraction index^40^. Co-contraction index was calculated for ankle joint (TA and MG) and knee joint (VL and BF) muscles and was normalized to the duration of each phase.

### Statistical analysis

A linear mixed-effect model using lower extremity (paretic, non-paretic, and non-dominant side in controls) as a fixed factor nested under a random factor of subject was used to test the differences of (1) peak ankle and knee joint rate of energy absorption, angular velocity, and torque during the impact absorption phase, and (2) ankle and knee joint muscle co-contraction index during the baseline and the impact absorption phase between groups. The p-values were adjusted using the Benjamini–Hochberg procedure^41^ to control the false discovery rate at 5% for multiple comparisons. Tukey’s HSD approach was performed to compare the estimated marginal means between each pair of groups. The significance level was set at an α of 0.05. Estimated marginal means are reported as ‘mean’ in the results. The percent difference between individuals post-stroke and the controls was calculated as the ratio of the difference of estimated marginal means between groups over the estimated marginal mean value of the control group.

If differences in the rate of energy absorption at the ankle or knee joint were detected, stepwise linear regression was performed to determine the relative contributions of the angular velocity and torque to the rate of energy absorption at the joint for each group. Simple linear regression was used to investigate the association between peak ankle rate of energy absorption during induced limb loading assessment and (1) self-selected and maximum walking speed, and (2) double support duration when the corresponding limb was the leading limb at self-selected and maximum speeds in individuals post-stroke. All the statistical analyses were conducted in R^42^.

## Results

**Figure 4:**
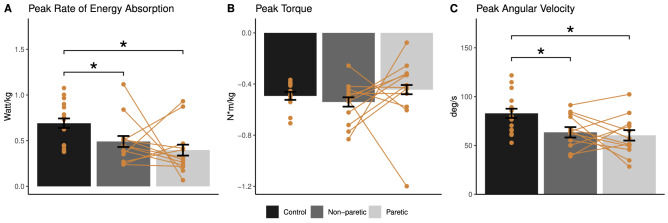
Between-group comparison of ankle joint kinetics and kinematics (mean ± standard error). Negative value indicates ankle plantarflexion in torque (**B**) and angular velocity (**C**). Yellow points show data from individual participants, and data from the same participant are linked with yellow lines. *p < 0.05, **p < 0.01.

**Figure 5:**
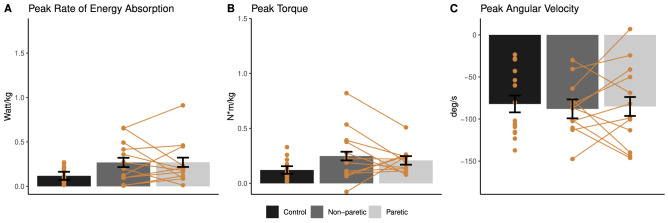
Between-group comparison of knee joint kinetics and kinematics (mean ± standard error). Negative value indicates knee flexion in torque (**B**) and angular velocity (**C**). Yellow points show data from individual participants, and data from the same participant are linked with yellow lines. *p < 0.05, **p < 0.01.

Reduced peak rate of energy absorption at the ankle joint was shown in both the paretic and non-paretic limb compared to controls (paretic: reduced by 42.5%, p = 0.002; non-paretic: reduced by 28.9%, p = 0.046; Fig. 4A). No group difference in peak ankle joint torque was found (p > 0.05; Fig. 4B). Both the paretic and non-paretic ankle joint showed reduced angular velocity compared to controls (paretic: reduced by 27.2%, p = 0.010; non-paretic: reduced by 23.4%, p = 0.028; Fig. 4C). No significant difference in knee joint rate of energy absorption, torque, and angular velocity between groups was found (Fig. 5).

Stepwise linear regression showed that ankle dorsiflexion angular velocity (β = 0.67, p < 0.001) contributed more to rate of energy absorption than joint torque (β = 0.48, p < 0.001) for the control group. In contrast, ankle joint torque was the primary contributor to rate of energy absorption for both the paretic and non-paretic limb (paretic: torque β = 0.84, p < 0.001, angular velocity β = 0.52, p < 0.001; non-paretic: torque β = 0.77, p = 0.004, angular velocity p > 0.05).

**Figure 6:**
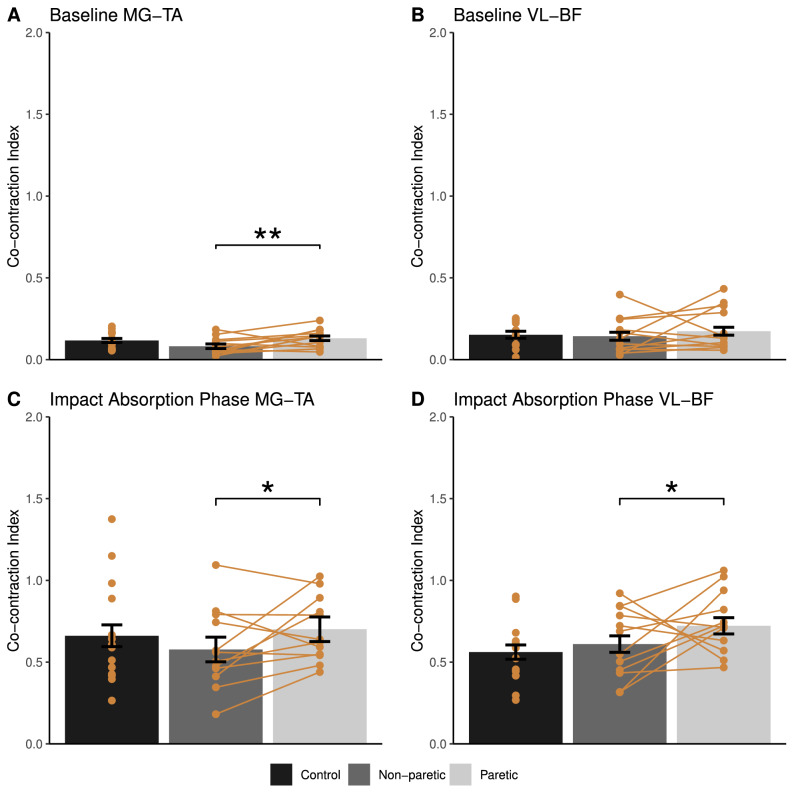
Between-group comparison of (**A**) Medial Gastrocnemius (MG) and Tibialis Anterior (TA) muscle co-contraction index during baseline, (**B**) Vastus Lateralis (VL) and Bicep Femoris (BF) muscle co-contraction index during baseline, (**C**) MG and TA muscle co-contraction index during the impact absorption phase, and (**D**) VL and BF muscle co-contraction index during the impact absorption phase (mean ± standard error). Yellow points show data from individual participants, and data from the same participant are linked with yellow lines. *p < 0.05, **p < 0.01.

No differences in co-contraction index were detected between individuals post-stroke and controls in ankle and knee joints. Higher co-contraction index during the impact absorption phase was found at the ankle and knee joints in the paretic side compared to the non-paretic side in individuals post-stroke (p = 0.01 for ankle, p = 0.04 for knee; Fig. 6C,D). Higher co-contraction index during baseline was also found at the paretic ankle but not the knee joint compared to the non-paretic side (p < 0.01; Fig. 6A,B).

**Figure 7:**
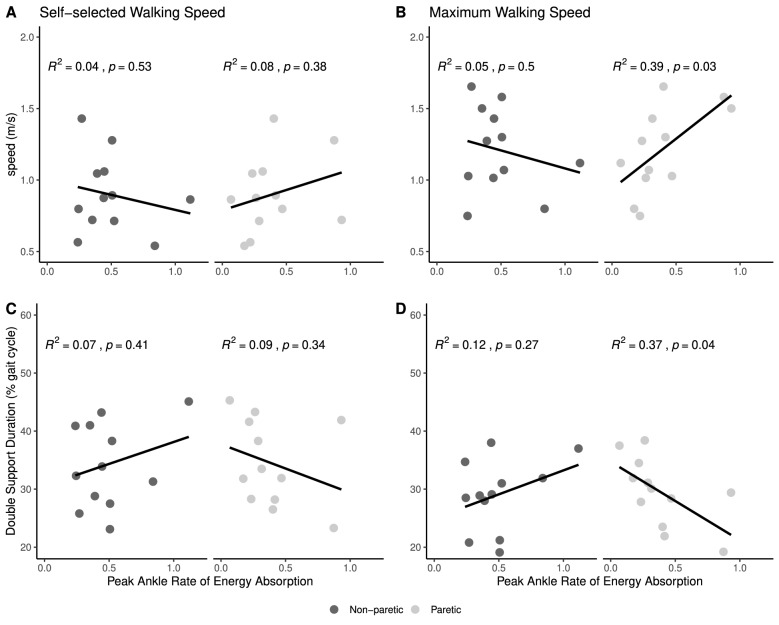
Relationships between peak ankle rate of energy absorption during imposed limb loading assessment and self-selected walking speed (**A**), maximal walking speed (**B**), double support duration during self-selected walking speed (**C**), and double support duration during maximal walking speed (**D**).

Peak paretic ankle rate of energy absorption during induced limb loading was correlated with walking speed (R^2^ = 0.39, p = 0.029) and double support duration (R^2^ = 0.37, p = 0.036) when walking at maximum walking speed in participants with chronic stroke. No correlation was found between peak paretic ankle rate of energy absorption and speed or double support duration when walking at a self-selected speed. No significant correlation was found between peak ankle rate of energy absorption at the non-paretic limb and speed or double support duration when walking with self-selected or maximum walking speed (Fig. 7).

## Discussion

In the present study, we assessed the biomechanical and neuromuscular factors that affect impact energy absorption during perturbation-induced limb loading in individuals post-stroke versus age-matched healthy controls. Our results revealed a reduced peak rate of energy absorption in the ankle joint in both paretic and non-paretic limbs among individuals post-stroke compared to controls, likely due to the limited dorsiflexion velocity at the ankle joint during the induced limb loading. In addition, individuals post-stroke who had higher rate of energy absorption at the paretic ankle joint during induced limb loading assessment also had higher speed and shorter double support duration when walking at maximum speed. These findings extend previous work^28^ by demonstrating that deficits in ankle dorsiflexion velocity during limb loading may limit the maximum mechanical energy absorption capacity of the joint and thereby affect weight transfer process and maximum walking performance following stroke.

Our findings showed that when undergoing rapid loading, both paretic and non-paretic ankle joints had a reduced rate of energy absorption compared to controls. These results were in contrast to a previous gait study that reported a higher rate of energy absorbed by the leading paretic limb ankle compared to healthy controls during step-to-step transition^43^. During gait, the energy absorbed by the leading limb is dependent on the push-off capacity of the contralateral trailing limb^44^. Because a higher rate of energy produced by the non-paretic trailing limb was reported compared to unimpaired controls in the study by Farris et al.^43^, the observed increases in the paretic ankle rate of energy absorption might have reflected the greater push-off power from the non-paretic trailing limb. In the present study, limb loading perturbations were delivered separately to each lower extremity, which allowed us to examine the rapid loading response of each limb in a more independent manner. With this approach, the impaired impact absorption ability in both paretic and non-paretic ankle joints was identified.

Consistent with a previous study that reported more energy absorbed at the paretic ankle joint than the knee joint during step-to-step transition in gait after stroke^43^, our result suggested that the ankle joint plays an important role in energy absorption during weight transfer. In addition, increased double support duration and decreased walking speeds are commonly observed following stroke^45^. Our result showed that participants with a higher energy absorption capacity at the paretic ankle joint during induced limb loading also had a shorter double support phase with the paretic limb as the leading limb during maximal walking speed, suggesting that greater paretic ankle energy absorption capacity could potentially contributed to more efficient weight transfer during locomotion. Moreover, participants with greater paretic ankle rate of energy absorption capacity also demonstrated greater maximum walking speed. These findings suggest that a deficit in the rate of energy absorption at the ankle joint may be an important target for rehabilitation that aims to improve limb loading and weight transfer efficiency post-stroke, especially for those stroke survivors with slower maximum walking speed. However, it is worth noting that the causation between paretic ankle rate of energy absorption and walking performance in individuals post-stroke remains to be determined. Future studies will investigate whether improving energy absorption capacity could lead to improvement in walking performance.

We did not find a significant relationship between ankle rate of energy absorption during induced limb loading assessment and self-selected walking speed in people post-stroke. This could be due to the different limb loading rate between tasks. During the induced limb loading, the assessed limb was loaded with an average of 98% bodyweight within 230 ms, which was about 20% shorter than the time to reach similar limb loading during walking at an average speed of 0.88 m/s in people post-stroke^46^. When walking with self-selected speed, the demand for energy absorption is submaximal during weight transfer, whereas walking at maximum speed likely demands greater energy absorption capacity of the paretic ankle. Given that the loading rate during induced limb loading assessment was much larger than that during walking under self-selected speed, the biomechanical characteristic identified during induced limb loading may be more reflective of the participants’ maximum capacity rather than preferred movement speed used in gait. Previous studies aiming to identify mechanisms that associate with maximum walking speed post-stroke have primarily focused on the power and force generation capacity at the paretic limb^47,48^. Our results expand upon these previous studies by demonstrating that, in addition to decreased force generation, reduced energy absorption ability could be a limiting factor for walking speed modulation capacity post-stroke.

Joint torque and angular velocity are two elements that contribute to the rate of energy absorption. We found that reduced dorsiflexion velocity at both the paretic and non-paretic ankle joint appeared to be the primary factor that limits rapid limb loading following stroke. The decreased joint velocity could be related to the exaggerated resistance to passive movement post-stroke^49^, which has been associated with hyperreflexia^50^, increased stiffness of the joint and muscle mechanical properties^51,52^, and increased muscle co-contraction^53^. Although the cause of reduced ankle dorsiflexion velocity in individuals post-stroke shown during the induced limb loading task could be the combination of all these factors, the weight of each factor could be different between the paretic and the non-paretic limb. We found that the co-contraction index was higher in the paretic ankle compared to the non-paretic side, indicating a higher contribution of the neuromuscular factor to the reduced dorsiflexion velocity at the paretic ankle. It is important to note that we also found a higher baseline co-contraction index in the paretic ankle compared to the non-paretic side. Thus, our results may reflect that abnormal baseline muscle activity could carry over to the impact absorption phase. Participants with stroke used ankle joint torque production as the primary strategy to absorb energy, rather than using ankle angular velocity as seen in able-bodied individuals. This altered strategy could indicate that individuals post-stroke utilized their retained ankle plantarflexion torque to compensate for deficits in ankle joint movement velocity to regulate impact absorption. Interventions that incorporate rapid ankle movements to enhance rate of energy absorption are likely important for restoring limb loading ability for individuals post-stroke. Importantly, reduced rate of energy absorption and dorsiflexion velocity at the ankle joint were observed in both the paretic and the non-paretic limbs compared to healthy controls. Thus, motor recovery evaluation and limb loading training for both limbs should be considered in the post-stroke rehabilitation program.

Previous studies have reported that muscle strength during isometric and concentric contraction is significantly reduced after stroke^54–56^. Results from the present study showed that ankle plantarflexion torque appeared to be preserved in participants with stroke during impact absorption. This discrepancy could be due to different approaches used across studies. In this study, the ankle plantarflexor muscles performed an eccentric contraction with a high velocity during rapid limb loading. Compared to a concentric contraction, joint torque production was less impaired during eccentric motion in individuals post-stroke, especially at higher speeds^56^. In addition to the contraction type, the joint position could also contribute to the preserved ankle plantarflexion torque in people post-stroke during induced limb loading. Previous studies have shown that the higher reflex-mediated and intrinsic muscle stiffness in the paretic ankle plantar flexors is most prominent when assessed in a dorsiflexed position^57^. During induced limb loading assessment, the ankle joint was dorsiflexed during the impact absorption phase, which could lead to a relatively higher plantarflexion torque compared to other studies.

Although we found a higher agonist–antagonist co-contraction at the paretic knee joint compared to non-paretic side, we did not identify any difference in knee joint kinetics and kinematics between groups. This is likely in part due to the task performed in this study. During vertical limb loading movements, the ankle has been reported to be the primary shock absorbing joint^58^. Our results support these previous findings by showing that abnormalities in the ankle joints are likely primary limiting factors of vertical limb loading ability following stroke. It is worth noting that in gait studies, the knee joint was reported to be the main impact absorber during normal walking^59^. A major difference between the present study and gait studies is the body configuration during limb loading. In the present study, participants were instructed to stand with a natural parallel stance. During post analysis, we observed that the ground reaction force vectors were oriented closely to the knee joint center of the loaded limb and therefore had a relatively small external moment arm and external torque applied to the knee joint. During dynamic gait, the leading limb is normally positioned in front of the body center of mass during initial contact and the ground reaction force vector is further away from the knee joint center. Thus, greater external knee torque was observed during walking compared to the present study^60^. These results warrant future studies to investigate the response to limb loading perturbation in different postures during gait. In particular, imposing limb loading to the leading limb during the double support phase of gait could induce ground reaction forces in the anterior–posterior direction and likely requires increased knee joint torque production and energy absorption.

There are limitations in this study. First, the perturbation was a relatively small vertical displacement of standing support surface to ensure participants’ safety. Although the perturbation intensity was sufficient to induce loading with an average of 98% bodyweight to the perturbed limb and identify neuromechanical abnormalities during limb loading, our results may not be generalizable to tasks with different intensities. Thus, further testing of a broader range of intensities may generate additional useful information. Secondly, only spatiotemporal gait characteristics were captured in this study. Although we found that deficits in ankle energy absorption capacity during induced limb loading were associated with longer double support duration during gait, the direct relationship between abnormalities in ankle and knee joint mechanics during gait versus induced limb loading remains to be determined. In addition, the reactive limb loading response tested in the present study could be inherently different from proactive limb loading during gait. To better understand whether our findings could be directly applicable to gait, future studies investigating forced limb loading during continuous gait is warranted. Last but not least, we did not include participants who wore an AFO during the imposed limb loading task in the final result. In the post analysis, we found that including participants with AFO produced results that were generally consistent with the current result, with reduced p-values in between-group comparisons of ankle power and angular velocity. However, these results are likely in part due to the reduced joint motion that is limited by the AFO. This additional confounding factor would hinder our ability to interpret the findings and focus on biological factors that affect energy absorption in the present study. Thus, participants wore AFO were excluded from the final analysis. Nonetheless, given that AFO is commonly worn by individuals with chronic stroke, future research investigating how AFO affects impact absorption and limb loading is warranted.

In conclusion, we studied the neuromechanical factors underlying impaired limb loading in individuals with chronic stroke versus able-bodied controls. We found that reduced ankle dorsiflexion velocity and reduced rate of energy absorption are likely key factors that limit rapid limb loading capacity following stroke. In addition, those with reduced rate of energy absorption at the paretic ankle joint revealed by induced limb loading assessment had a prolonged double support duration and decreased gait velocity when walking at maximum speed. Future research will determine the effectiveness of repetitive limb loading perturbation training on improving limb loading, impact absorption, and walking ability post-stroke.

## Data Availability

The datasets generated and analyzed during the current study are available from the corresponding author on reasonable request.

## Abbreviations

BF: Biceps femoris
COP: Center of pressure
EMG: Electromyography
GM: Gluteus medius
GRF: Ground reaction force
MG: Medial gastrocnemius
TA: Tibialis anterior
HSD: Honestly significant difference
